# A study of auxiliary screening for Alzheimer’s disease based on handwriting characteristics

**DOI:** 10.3389/fnagi.2023.1117250

**Published:** 2023-03-15

**Authors:** Hengnian Qi, Ruoyu Zhang, Zhuqin Wei, Chu Zhang, Lina Wang, Qing Lang, Kai Zhang, Xuesong Tian

**Affiliations:** ^1^Information Engineering Department, Huzhou University, Huzhou, China; ^2^School of Medicine and Nursing, Huzhou University, Huzhou, China; ^3^Library, Huzhou University, Huzhou, China; ^4^School of Information Engineering, Guangdong Communication Polytechnic, Guangzhou, China; ^5^Cloudbutterfly Technology Co., Ltd., Guangzhou, China

**Keywords:** Alzheimer’s disease, handwriting characteristic, auxiliary screening, auxiliary diagnosis, classification models

## Abstract

**Background and objectives:**

Alzheimer’s disease (AD) has an insidious onset, the early stages are easily overlooked, and there are no reliable, rapid, and inexpensive ancillary detection methods. This study analyzes the differences in handwriting kinematic characteristics between AD patients and normal elderly people to model handwriting characteristics. The aim is to investigate whether handwriting analysis has a promising future in AD auxiliary screening or even auxiliary diagnosis and to provide a basis for developing a handwriting-based diagnostic tool.

**Materials and methods:**

Thirty-four AD patients (15 males, 77.15 ± 1.796 years) and 45 healthy controls (20 males, 74.78 ± 2.193 years) were recruited. Participants performed four writing tasks with digital dot-matrix pens which simultaneously captured their handwriting as they wrote. The writing tasks consisted of two graphics tasks and two textual tasks. The two graphics tasks are connecting fixed dots (task 1) and copying intersecting pentagons (task 2), and the two textual tasks are dictating three words (task 3) and copying a sentence (task 4). The data were analyzed by using Student’s *t*-test and Mann–Whitney U test to obtain statistically significant handwriting characteristics. Moreover, seven classification algorithms, such as eXtreme Gradient Boosting (XGB) and Logistic Regression (LR) were used to build classification models. Finally, the Receiver Operating Characteristic (ROC) curve, accuracy, sensitivity, specificity, positive predictive value (PPV), negative predictive value (NPV), and Area Under Curve (AUC) were used to assess whether writing scores and kinematics parameters are diagnostic.

**Results:**

Kinematic analysis showed statistically significant differences between the AD and controlled groups for most parameters (*p* < 0.05, *p* < 0.01). The results found that patients with AD showed slower writing speed, tremendous writing pressure, and poorer writing stability. We built statistically significant features into a classification model, among which the model built by XGB was the most effective with a maximum accuracy of 96.55%. The handwriting characteristics also achieved good diagnostic value in the ROC analysis. Task 2 had a better classification effect than task 1. ROC curve analysis showed that the best threshold value was 0.084, accuracy = 96.30%, sensitivity = 100%, specificity = 93.41%, PPV = 92.21%, NPV = 100%, and AUC = 0.991. Task 4 had a better classification effect than task 3. ROC curve analysis showed that the best threshold value was 0.597, accuracy = 96.55%, sensitivity = 94.20%, specificity = 98.37%, PPV = 97.81%, NPV = 95.63%, and AUC = 0.994.

**Conclusion:**

This study’s results prove that handwriting characteristic analysis is promising in auxiliary AD screening or AD diagnosis.

## 1. Introduction

According to the World Health Organization (WHO), the elderly population is growing rapidly and will reach nearly 1.4 billion by 2030 and increase to nearly 2.1 billion by 2050 (Ammal and Manoharan, 2023). Population aging has promoted researches on age-related diseases and mental diseases (Gaugler et al., 2022). Alzheimer’s disease is the most common form of dementia and is becoming increasingly common (Thies and Bleiler, 2012; White et al., 2019). According to the latest statistics from the Centers for Disease Control and Prevention (CDC), the number of people with AD doubles every 5 years. According to the World Health Organization, it is estimated that the number of people with AD will reach almost 152 million by 2050 (Lei et al., 2021). It is characterized by a gradual onset of symptoms and an irreversible decline to a near vegetative state, with an average survival period of about 10 years (Small et al., 1997; Edition, 2013). After Alzheimer’s disease reaches the dementia stage, it is currently medically incurable, and the only available pharmacological treatment is to slow the progression (Kverno, 2022), making early screening of AD particularly important.

Writing is a complex human activity that requires a complex mixture of cognitive, kinesthetic, and perceptual-motor components (Hunter,1974; Kverno, 2022), which includes visual and kinesthetic perception, motor planning, eye-hand coordination, visuomotor integration, dexterity, and manual skills (Tseng and Cermak, 1993). Also, writing is one of the abilities affected by Alzheimer’s disease (Platel, 1993; Tseng and Cermak, 1993; Hughes et al., 1997; Forbes et al., 2004; Caterina Silveri et al., 2007; Cilia et al., 2019). Indeed, when signs of the disease become apparent, the impairment may already have been significant and irreversible. In this context, it is widely accepted that writing is one of the first skills affected by cognitive impairment (Pereira et al., 2018). There is evidence that a healthy or unhealthy person can be distinguished by his or her ability to perform simple writing activities (Kawa et al., 2017), so some differences in writing handwriting affected by cognitive disorders may provide some basis for diagnosing these disorders.

So far, most of the studies on handwriting in AD have been conducted in the field of medicine and psychology, which typically use statistically relevant methods to find some statistically significant features of the patient’s handwriting (Stefano et al., 2018). It is also concluded that handwriting is a good alternative biomarker for assessing AD, and that handwriting analysis research is considered promising (Impedovo and Pirlo, 2018). In addition, with the popularization and development of computers, computers, and artificial intelligence have been carried out in corresponding fields, mainly using various algorithms to screen features and build the classification model (Stefano et al., 2018; Dentamaro et al., 2021). Some researchers use dynamic features in combination with images and find that dynamic information can improve the performance of binary images (Cilia et al., 2021). And have achieved good results by using machine learning algorithms (Cilia et al., 2022). These studies have detected AD patients with mild motor dysfunction (Ghilardi et al., 2000; Smits et al., 2014), with the most common abnormalities being micro writing, slower movement, and other (Smits et al., 2014).

The purpose of this study is to conduct an exploratory study on the value of AD-assisted diagnosis based on the kinematic characteristics of different tasks. This study considers a variety of handwriting tasks that examine cognitive states. Four handwriting tasks were finally designed to construct a set of writing characteristics applicable to distinguish the handwriting of the AD group from that of healthy controls. In order to better reproduce handwriting, a dot-matrix pen and paper were used to replace the digitizing tablet device commonly used to capture handwriting in the past. Using the “Huzhou Normal University Handwriting characteristic Analysis System (huSaS)” based on dot matrix paper and pen developed by this team, the handwriting characteristics of 34 AD patients and 45 healthy controls were automatically and quantified by pattern recognition technology, and the differences in handwriting characteristics between AD group and healthy control group were analyzed according to the obtained data. The resulting data were used to analyze the differences between the handwriting characteristics of the AD group and the healthy controls.

## 2. Materials and methods

### 2.1. Participants

The recruitment period is from September 2021 to January 2022. Seventy-nine outpatients were recruited from the geriatric psychiatric outpatient clinic of the Third People’s Hospital of Huzhou City, China. Of these, 34 with probable AD, and 45 healthy seniors, took part in this experiment. The diagnosis of AD was based on medical records, psychometric testing, neuroimaging, and follow-up investigations. All fulfilled the criteria specified for probable AD (McKhann et al., 2011). All of them were mild AD patients. All participants performed the Mini-Mental State Examination (MMSE; Folstein et al., 1975) and the Montreal Cognitive Assessment Basic (MoCA-BC; Chen et al., 2016) at the same day as the assessment of handwriting. Obtaining permission is shown in Supplementary Figure S1. All participants used right hands as their dominant hands, and their native language was Chinese. And the exclusion criteria are as follows: psychiatric disorders (e.g., depression, manic-depressive psychosis, etc.), history of substance abuse (e.g., alcoholism), head injury including loss of consciousness, or any other additional sensory condition that may adversely affect the mental status or motor function (e.g., visual impairment unless normalized with spectacle correction), developmental learning disabilities. A summary of the demographic characteristics of the participants is given in Table 1.

**Table 1:** Comparison of general information of AD patients and the control group.

Variable	AD (*n* = 34)	HC (*n* = 45)	*z* value/ χ^2^ value	Value of *p*
Gender (*n*, %)			0.001	0.977
Male	15 (44.1)	20 (44.4)		
Female	19 (55.9)	25 (55.6)		
Age (years, $\bar{x}\pm s$ )	77.15 ± 1.796	74.78 ± 2.193	−0.743	0.457
Years of education (years, $\bar{x}\pm s$ )	7.79 ± 0.588	8.91 ± 0.575	−1.852	0.064
MMSE (score, $\bar{x}\pm s$ )	18.21 ± 0.695	27.93 ± 0.245	−7.355	<0.001
MoCA-BC (score, $\bar{x}\pm s$ )	14.88 ± 0.732	28.93 ± 0.245	−7.661	<0.001
Nature of work (*n*, %)			0.560	0.454
Mental work	16 (47.1)	25 (55.6)		
Physical labor	18 (53.0)	20 (44.4)		
Residence status (*n*, %)			5.513	0.239
Other	6 (17.7)	2 (4.5)		
Live with spouse	16 (47.1)	29 (64.5)		
Live with spouse and children	1 (2.9)	3 (6.7)		
Living alone	5 (14.7)	6 (13.3)		
Live with children	6 (17.6)	5 (11.1)		
Area of residence (*n*, %)			0.002	0.967
City	21 (61.8)	28 (62.2)		
Rural	13 (38.2)	17 (37.8)		
Monthly income (*n*, %)			−0.490	0.624
≤1,000 RMB	8 (23.6)	9 (20.0)		
1,001–2,999 RMB	8 (23.6)	10 (22.2)		
3,000–4,999 RMB	5 (14.7)	9 (20.0)		
≥5,000 RMB	12 (35.3)	17 (37.8)		

AD, Alzheimer’s disease; HC, healthy control participants; MMSE, mini-mental state examination; MoCA-BC, montreal cognitive assessment basic.

This study was approved by the Medical Ethics Committee of the Third People’s Hospital of Huzhou, China (review number: 2022–049) and obtained the informed consent of all participants.

### 2.2. Methods

#### 2.2.1. Selection of handwriting acquisition tools

**Figure 1:**
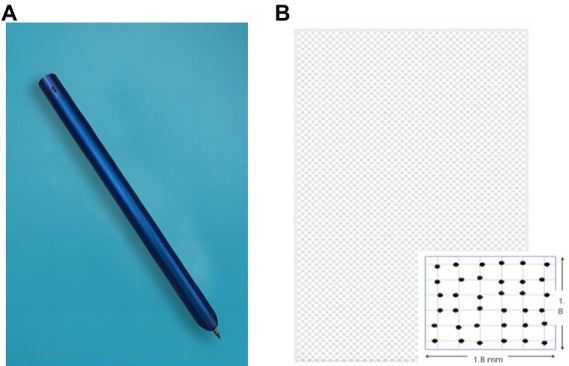
**(A)** Dot-matrix pen diagram; **(B)** Dot-matrix paper diagram.

In order to make handwriting acquisition more convenient, considering that writing with paper and pen is more suitable for the writing habits of the elderly and controlling irrelevant variables, the dot-matrix digital pen was selected as the handwriting acquisition tool in this study. After careful consideration and screening, the dot-matrix pen (TSTUDY, China) was finally selected as the writing tool in this study, which mainly consists of a pressure sensor, a high-speed camera, a processor, a battery, data memory, and a Bluetooth or USB communication module. The pressure sensor (pressure sensing level 1,024) is triggered when the pen tip is pressed down. The built-in high-speed camera is activated to take pictures of the dots passed by the pen tip at 100 frames per second. The information such as dots coordinates, handwriting order, pressure data, and movement speed are transmitted to the built-in processor, which is finally output to the outside *via* Bluetooth or USB communication unit. The “dot-matrix” is composed of some tiny dots arranged according to special algorithm rules, which can provide the digital pen with a piece of coordinate parameter information to accurately record the digital pen writing on the digital handwriting by printing on ordinary paper (through ordinary laser printers, professional printing) on a layer of dot-matrix pattern, you can make paper becomes intelligent. Furthermore, the dot-matrix pen supports secondary application development after obtaining authorization, providing the possibility of obtaining more writing characteristics. Figure 1A shows a picture of the dot-matrix pen, and Figure 1B shows the dot-matrix data unit.

#### 2.2.2. Selection of handwriting acquisition tools

According to the characteristics of writing tasks selected by previous authors (Luzzatti et al., 2003; Schrter et al., 2003; Werner et al., 2006; Evyapan Akku, 2015; Garre-Olmo et al., 2017; Kawa et al., 2017; Kahindo et al., 2018), combined with the needs of this study, after repeated demonstrations by our team, the selection criteria of the writing tasks were finally determined: the investigation is comprehensive, moderate difficult, easy to understand, and emotionally neutral. After pre-experimentation, the following four handwriting tasks (two graphic and two textual tasks) were finally selected: (1) Connection of fixed points. Four dots were connected as required, and the shapes of the two groups were “十” and “X” respectively. This task examines the participants’ fine control and execution of the wrists. (2) Copying the intersecting pentagon. This task required drawing the two intersecting pentagons given. This task examined the ability to copy geometric figures and to perform two-dimensional visual space. (3) Dictate three Chinese words: “儿子,” “女儿,” “大米,” which means: “son,” “daughter,” and “rice,” pronounced “Er Zi,” “Nu Er,” and “Da Mi.” This task was designed to examine the participants’ short-term memories and executive abilities. (4) Copy a phrase. The participants were asked to copy a Chinese sentence: “开心每一秒，快乐每一天,” which means: “Happy every second, pleasure every day.” This task was designed to examine spatial organization and execution skills. The writing task is shown in Supplementary Figure S1.

#### 2.2.3. Screening of handwriting characteristic

This study was based on the handwriting characteristics extracted from previous studies and finally screened 33 objective handwriting characteristics: two items in the time category (time in the air, single stroke time); seven items in the length category [single stroke length, horizontal/vertical stroke length, horizontal/vertical stroke length (variance, standard deviation)]; four items in the pressure category (average mean, variance, standard deviation, and entropy value); 17 items in the speed category (velocity entropy value, average velocity, and horizontal/vertical average velocity, velocity maximum value, minimum value, variance, and standard deviation); the number of times the velocity slowed down, horizontal/vertical velocity (maximum value, minimum value, variance, and standard deviation); three items in the acceleration category (acceleration entropy value, number of times the acceleration slowed down); and two other items (number of strokes, tilt). The writing was also assessed for each handwriting by task using specific scoring criteria. The scoring criteria for task 1 were connecting orientation, and accurate angle, which satisfied counting one point for two groups and two points in total; the scoring criteria for task two was a pentagon, closed intersection, and roughly the same side length, which satisfied one point for one item and three points in total; the rest of the tasks were scored according to the correctness of each word, and one point for writing one word correctly. A total of 35 subjective handwriting characteristics and objective handwriting characteristics were finally obtained (Table 2).

**Table 2:** List of 35 handwriting characteristics.

No.	Characteristic name	Unit	Characteristic description
1, 4, and 5	Pressure (mean, variance, and standard deviation)	Level (0–1,024)	The mean of the pressure, the pressure variance, and the standard deviation of the pressure at the time of writing
2, 20, 32	Pressure/velocity/acceleration entropy	bit	#
3	Time in the air	ms	Pen movement time in the air
7	Single stroke time	ms	Paper and pen contact time
6	Tilt angle	°	The angle of inclination of the writing direction intersecting with the horizontal line
8, 9	Horizontal/vertical stroke length	mm	Horizontal/vertical writing length
10	Single stroke length	mm	Length when writing with a single stroke
11–14	Horizontal/vertical stroke length (variance, standard deviation)	mm	(variance, standard deviation) of writing length in horizontal/vertical direction
15	Average speed	mm/s	The average speed of writing
16, 17	Horizontal/vertical average speed	mm/s	The average speed of writing in horizontal/vertical direction
18,19, 22, and 23	Velocity (maximum value, minimum value, variance, and standard deviation)	mm/s	Write of speed (maximum, minimum, variance, standard deviation)
21, 33	Number of times velocity/acceleration slowed down	times	Number of times the speed/acceleration slows down while writing
24, 25, 28, 29, 26, 27, 30, and 31	Horizontal/vertical velocity (maximum value, minimum value, variance, and standard deviation)	mm/s	(maximum value, minimum value, variance, standard deviation) of horizontal/vertical velocity during the writing
34	Task score	score	Writing score under the corresponding evaluation criteria
35	Number of strokes	#	Number of separable strokes in the whole task

No., Characteristic number; ”#,” No units.

#### 2.2.4. Development of handwriting analysis system

In order to facilitate the collection, a handwriting collection APP is written in Android language to realize the visualization of the collection, in which the handwriting reproduction is convenient for the preliminary test to observe whether the handwriting is successfully collected and to delete the incorrect data in a single line or in a batch in time. Transmitting to the database SQLite on the computer side *via* Bluetooth, and the collected writing information can be automatically transformed into characteristic handwriting data.

#### 2.2.5. Handwriting acquisition procedure and data processing

Participants were invited to a quiet room and sat on a chair in front of a table. Dot-matrix pens and dot-matrix papers (210 mm × 297 mm) printed with four tasks were placed on the table, and the lower edge of the paper was aligned with the edge of the table where the participants were sitting. The participants’ forearms are placed at an angle of about 30 degrees to the lower edge of the paper. The experimenter read the instructions and demonstrated the writing process. The participants performed a 5-min breathing exercise (fine and deep long) before writing to ensure that the writing was done calmly, at a self-paced and comfortable speed.

Extract handwriting information in stroke units. Since Chinese characters are composed of multiple strokes, this study divides the writing handwriting into single strokes. The standard for splitting a single stroke is that the pen tip touches the paper until the pen is written again before the next stroke (A corresponding induction device inside the dot-matrix pen can record the contact state of the pen tip and the paper). Based on the handwriting acquisition system developed by the research group, using the code written for each feature, the collected basic position, time and pressure information are converted into the required handwriting feature values. Because each stroke was taken as an independent sample, the characteristics of each stroke were not averaged over the strokes.

**Figure 2:**
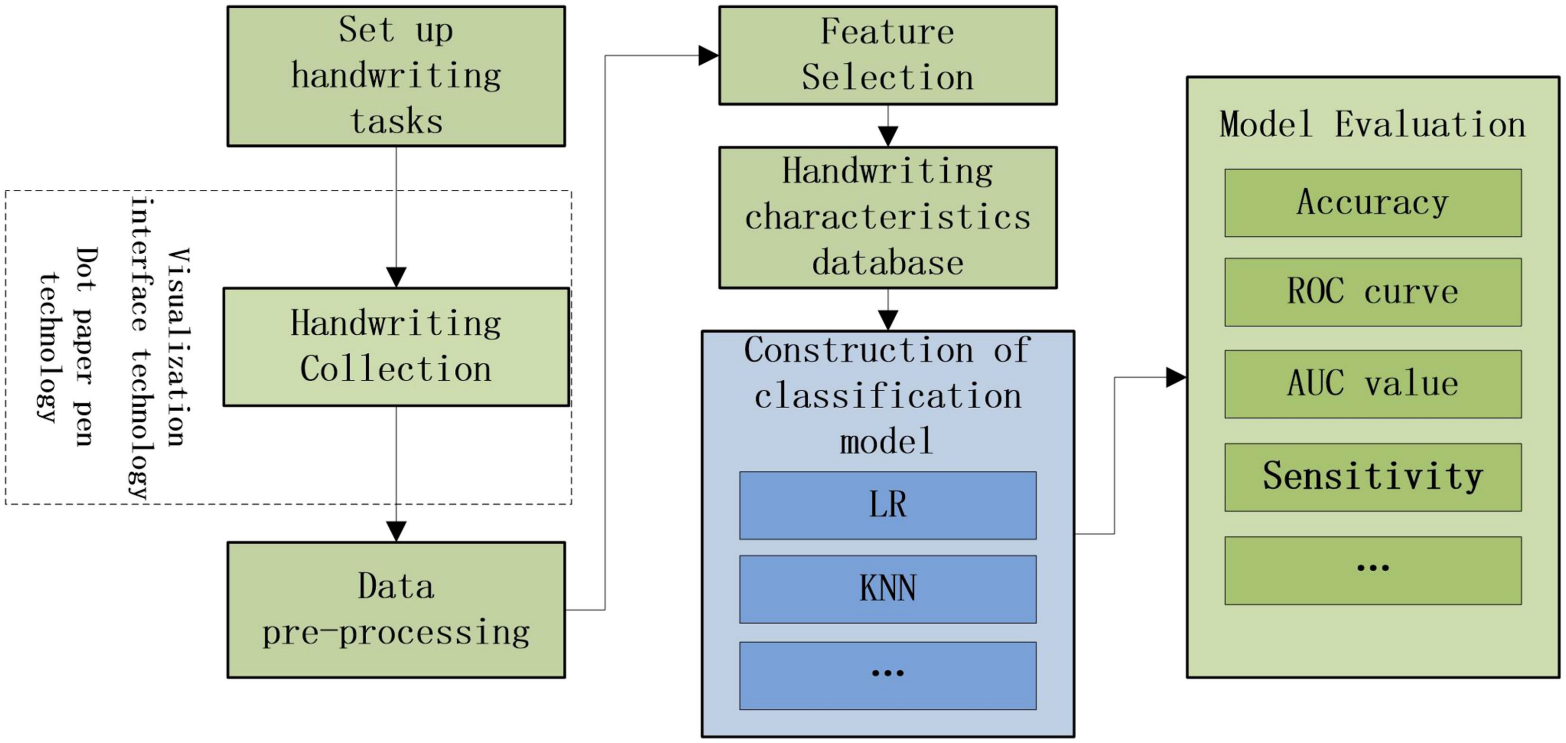
Experimental design scheme diagram.

Data preprocessing was first performed for the extracted data to fill in the missing data with the average mean. Then the obtained data are analyzed and selected for handwriting characteristics. And independent sample Student’s *t*-test and Mann–Whitney U-test were performed using SPSS26.0, respectively, according to whether the normality test and ANOVA were satisfied, and differences were considered statistically significant at *p* < 0.05. The above process obtains a handwriting characteristic database. PyCharm Community Edition 2020.3.2 ×64 was used for programming, using Logistic Regression (LR), K-Nearest Neighbors (KNN), Support Vector Machine (SVM), GaussianNB (GNB), Random Forest (RF), eXtreme Gradient Boosting (XGB), and Adaptive Boosting (Adaboost) to build classification models. For each ROC curve, we used Youden’s method to find the optimal threshold values as well as the Sensitivity (Se.), Specificity (Sp.), Positive Predictive Value (PPV), Negative Predictive Value (NPV), Accuracy (Acc.), and Area Under the Curve (AUC). The overall design scheme diagram is shown in Figure 2.

## 3. Results

In the general information of the patients and control groups (Table 1), the Mann–Whitney test showed significant differences between the two groups in MMSE (*p* < 0.001) and MoCA-BC (*p* < 0.001). In terms of gender (*p* = 0.977, Chi-square test), age (*p* = 0.457), years of education (*p* = 0.064), nature of work (*p* = 0.454, Chi-square test), residence status (*p* = 0.239, Chi-square test), area of residence (*p* = 0.967, Chi-square test), and monthly income (*p* = 0.624, Kruskal-Wallis test) were not significantly different.

Kinematic analysis showed significant differences between the AD and control groups on most parameters (Tables 3, 4). In task 1, there were statistically significant differences between AD patients and controls on 14 handwriting characteristics such as pressure variance (*p* < 0.05, *p* < 0.01); in task 2, there were statistically significant differences between AD patients and controls on 19 handwriting characteristics such as pressure average mean (p < 0.05,p < 0.01); in task 3, there were statistically significant differences between AD patients and controls on In task 3, there were statistically significant differences between AD patients and controls in 17 handwriting characteristics, including time in the air and single stroke time (*p* < 0.05, *p* < 0.01); in task 4, there were statistically significant differences between AD patients and controls in 14 handwriting characteristics, including the number of strokes and single stroke length (*p* < 0.05, *p* < 0.01).

**Table 3:** Student’s *t*-test summary (only parameters with *p* < 0.05).

No.	Task #	AD		HC		*t* value	Value of *p*	MoCA-BC correlation	
		Mean	Std	Mean	Std			Rs	*p*
5A	1	131.19	5.77	115.46	4.10	−2.95	0.003	**−1.31**	**0.009**
13E	1	88.51	7.08	130.76	7.44	3.67	0.004	**0.20**	**<0.001**

Statistically significative correlation to MoCA-BC results marked bold in columns (9–10). No., characteristic number; AD, Alzheimer’s disease; HC, healthy control participants; MoCA-BC, montreal cognitive assessment basic; and Std, standard deviation. Bold indicates that the results were significant. Units: A-pressure sensation (1,024 levels), E-mm.

**Table 4:** Mann–Whitney U test summary (only parameters with *p* < 0.05).

No.	Task #	AD		HC		*z* value	Value of *p*	MoCA-BC correlation	
		Median	IQR	Median	IQR			Rs	*p*
8E	1	7.42	15.84	19.31	17.93	−3.04	0.002	**0.20**	**<0.001**
10E	1	16.09	18.96	23.66	9.12	−4.37	<0.001	**0.24**	**<0.001**
14E	1	26.92	123.56	30.91	141.70	−2.23	0.026	0.16	0.002
15C	1	10.748	9.689	15.059	12.082	−4.35	<0.001	0.09	0.075
16C	1	7.38	9.57	10.50	12.56	−2.94	0.003	0.08	0.100
17C	1	5.93	5.97	6.74	11.21	−2.10	0.036	0.07	0.179
18C	1	43.63	38.29	59.32	42.67	−4.58	<0.001	**0.16**	**0.001**
23C	1	9.97	8.25	13.50	10.66	−4.25	<0.001	**0.12**	**0.019**
24C	1	31.91	30.99	46.25	36.31	−3.45	0.001	**0.13**	**0.012**
26C	1	25.44	27.20	29.60	38.54	−2.51	0.012	**0.15**	**0.003**
34H	1	1.00	2.00	2.00	0.00	−13.31	<0.001	**0.69**	**<0.001**
35I	1	5.00	1.00	5.00	2.00	−3.31	0.001	**−0.24**	**<0.001**
1A	2	661.09	238.31	579.10	362.69	−3.31	0.001	**−0.29**	**<0.001**
2B	2	4.16	1.23	4.39	1.09	−3.77	<0.001	0.11	0.078
5A	2	142.98	85.29	132.05	67.00	−2.72	0.007	**−0.15**	**0.018**
6D	2	−52.23	105.75	−34.09	103.30	−2.54	0.011	**−0.12**	**0.047**
8E	2	6.96	9.20	8.88	10.66	−2.72	0.007	0.06	0.329
9E	2	6.81	8.86	7.94	9.34	−2.07	0.039	0.03	0.596
10E	2	11.04	11.82	12.34	11.84	−2.54	0.011	0.05	0.438
13E	2	49.39	80.82	76.32	92.77	−4.62	<0.001	0.09	0.144
14E	2	50.38	71.95	67.31	70.48	−4.09	<0.001	0.12	0.055
15C	2	7.89	8.53	10.68	9.00	−4.99	<0.001	**0.25**	**<0.001**
16C	2	4.37	5.69	6.35	6.19	−4.54	<0.001	**0.19**	**0.002**
17C	2	4.63	5.48	6.14	5.67	−4.15	<0.001	**0.21**	**0.001**
18C	2	40.97	42.22	49.57	36.98	−3.39	0.001	**0.13**	**0.030**
20B	2	4.83	1.43	4.88	1.18	−1.36	0.173	0.02	0.766
23C	2	8.50	9.81	11.27	9.84	−4.90	<0.001	**0.15**	**0.012**
24C	2	27.75	35.61	31.14	30.83	−2.24	0.025	0.10	0.093
26C	2	27.75	27.13	33.30	31.80	−3.14	0.002	**0.13**	**0.032**
34H	2	11.00	9.00	10.00	5.00	−17.65	<0.001	**0.15**	**0.013**
35I	2	1.00	2.00	3.00	0.00	−4.56	<0.001	**−0.17**	**0.005**
1A	3	625.20	182.72	541.58	306.24	−8.14	<0.001	**−0.23**	**<0.001**
2B	3	3.73	1.03	3.46	1.00	−5.49	<0.001	**−0.17**	**<0.001**
3C	3	616.00	1320.00	246.00	512.00	−11.39	<0.001	**−0.16**	**<0.001**
7C	3	472.00	544.00	288.00	332.00	−10.28	<0.001	**−0.25**	**<0.001**
15C	3	16.51	15.40	26.78	19.13	−12.43	<0.001	**0.39**	**<0.001**
16C	3	8.71	10.72	13.88	13.63	−9.30	<0.001	**0.30**	**<0.001**
17C	3	9.73	11.95	16.91	16.73	−11.20	<0.001	**0.33**	**<0.001**
18C	3	52.78	41.66	71.21	48.42	−9.50	<0.001	**0.27**	**<0.001**
20B	3	4.11	1.38	3.58	1.32	−6.67	<0.001	**−0.20**	**<0.001**
21G	3	8.00	11.00	5.00	7.00	−7.92	<0.001	**−0.24**	**<0.001**
23C	3	13.53	13.16	20.91	15.38	−10.53	<0.001	**0.29**	**<0.001**
24C	3	32.38	35.15	44.40	38.85	−6.57	<0.001	**0.22**	**<0.001**
26C	3	37.00	34.69	51.80	48.10	−8.42	<0.001	**0.23**	**<0.001**
32B	3	4.25	1.54	3.70	1.46	−7.56	<0.001	**−0.24**	**<0.001**
33G	3	9.00	11.00	6.00	8.00	−7.51	<0.001	**−0.23**	**<0.001**
34H	3	6.00	2.00	6.00	0.00	−16.73	<0.001	**0.37**	**<0.001**
35I	3	19.00	5.00	18.00	3.00	−8.03	<0.001	**−0.45**	**<0.001**
1A	4	620.65	175.93	523.64	298.39	−13.44	<0.001	**−0.43**	**<0.001**
2B	4	3.66	1.05	3.17	1.02	−13.28	<0.001	**−0.26**	**<0.001**
3C	4	502.00	1161.00	180.00	320.00	−20.99	<0.001	**−0.27**	**<0.001**
7C	4	412.00	513.00	220.00	244.00	−18.48	<0.001	**−0.23**	**<0.001**
10E	4	6.68	6.10	5.84	5.75	−2.40	0.016	**−0.35**	**<0.001**
13E	4	25.39	37.50	27.87	30.12	−0.54	0.587	**−0.23**	**<0.001**
18C	4	49.92	39.82	65.17	46.85	−12.83	<0.001	**−0.35**	**<0.001**
20B	4	3.88	1.39	3.17	1.42	−14.27	<0.001	**−0.25**	**<0.001**
21G	4	7.00	10.00	4.00	6.00	−16.12	<0.001	**−0.21**	**<0.001**
23C	4	13.39	11.65	19.38	14.43	−15.24	<0.001	**−0.35**	**<0.001**
24C	4	29.60	36.08	41.63	41.75	−10.67	<0.001	**−0.28**	**<0.001**
26C	4	32.38	35.65	43.94	46.25	−9.50	<0.001	**−0.35**	**<0.001**
32B	4	4.03	1.61	3.32	1.50	−16.16	<0.001	**−0.25**	**<0.001**
33G	4	8.00	11.00	4.00	6.00	−16.25	<0.001	**−0.23**	**<0.001**

Statistically significative correlation to MMSE results marked bold in columns (9–10). No., characteristic number; AD, Alzheimer’s disease; HC, healthy control participants; MoCA-BC, montreal cognitive assessment basic; and IQR, inter quartile range. Bold indicates that the results were significant. Units: A-pressure sensation (1,024 levels), B-bit, C-ms, D-°, E-mm, E-mm, F-m/s, G-bit, H-score, and I-#.

Through observation, we found that each task’s AD group and the control degree had some characteristics in common. For example, in each task, the maximum speed per stroke, the maximum velocity in the horizontal and vertical directions, and the standard deviation of the velocity in the AD group were smaller than that in the control group.

In addition to the common characteristics, we found that the handwriting characteristics of the two groups showed some subtle differences in different tasks. In Task 1, the variance and standard deviation of pressure in the AD group were higher than those in the control group, indicating that AD patients had poor stability of pressure when performing Task 1; the number of strokes did not differ between the two groups; the remaining variables showed smaller values in the AD group. In task 2, the AD group’s task score was not lower than that of the control group; the average mean, standard deviation, and variance of pressure in the AD group were higher than those of the control group, indicating that in task 2, the AD patients wrote with more pressure and were more unstable. In tasks 3 and 4, the pressure entropy value, velocity entropy value, acceleration entropy value, number of slowed-down velocities, number of strokes, and number of slowed-down accelerations were all higher than those of the control group, indicating that AD patients were less stable in writing; the average mean value of pressure, single stroke time, and time in the air were all higher than those of the control group, indicating that AD patients were more pressured and acted less responsively when writing, requiring more writing time and time for reflection and recall. There were no differences in task scores in task 3; the remaining variables showed smaller values for the AD group. In task 4, the single stroke length was longer in the AD group, indicating a larger writing font; the remaining variables showed smaller values in the AD group.

Several characteristics showed statistically significant (*p* < 0.05), primarily negative, correlation with the reference MoCA-BC results: results marked bold in Tables 3, 4.

Entering all features into the classifier, the classification is not satisfactory. So, the statistically significant features (all features are included in Tables 3, 4 without further optimization) were used as inputs in seven classifiers to test the effectiveness of the classification. These seven classifiers are Logistic Regression (LR), K-Nearest Neighbors (KNN), Support Vector Machine (SVM), GaussianNB (GNB), Random Forest (RF), eXtreme Gradient Boosting (XGB), and Adaptive Boosting (Adaboost). Tables 5–8 show each classifier’s accuracy, specificity, sensitivity, positive predictive amount, negative predictive amount, and AUC values. The results show that the accuracy of each model, except the KNN classifier in task 3, all ranged from 71.5 to 96.55%. The AUC values ranged from approximately 0.75–0.99, which achieved good results, indicating that the written assessment and kinematic parameters were diagnostic aids in distinguishing the AD group from the control group.

**Table 5:** Performance of classifier implementation on task 1.

	LR	KNN	SVM	GNB	RF	XGB	Adaboost
Acc. (%)	77.78%	73.50%	76.92%	72.65%	77.78%	83.76%	77.78%
Se (%)	80.00%	58.33%	65.00%	53.33%	75.00%	81.67%	65.00%
Sp (%)	75.44%	89.47%	89.47%	92.98%	80.70%	85.96%	91.23%
PPV (%)	77.42%	85.37%	86.67%	88.89%	80.36%	85.96%	88.64%
NPV (%)	78.18%	67.11%	70.83%	65.43%	75.41%	81.67%	71.23%
AUC	0.856	0.761	0.835	0.768	0.849	0.891	0.839
Optimal threshold	0.335	0.600	0.598	1.000	0.474	0.379	0.962

Se, sensitivity; Sp, specificity; PPV, positive predictive value; NPV, negative predictive value; Acc., accuracy; and AUC, area under the curve.

**Table 6:** Performance of classifier implementation on task 2.

	LR	KNN	SVM	GNB	RF	XGB	Adaboost
Acc. (%)	91.36%	77.78%	86.42%	82.72%	91.98%	96.30%	95.68%
Se (%)	80.28%	70.42%	81.69%	77.46%	84.51%	100.00%	95.77%
Sp (%)	100.00%	83.52%	90.11%	86.81%	97.80%	93.41%	95.60%
PPV (%)	100.00%	76.92%	86.57%	82.09%	96.77%	92.21%	94.44%
NPV (%)	86.67%	78.35%	86.32%	83.16%	89.00%	100.00%	96.67%
AUC	0.932	0.834	0.900	0.859	0.929	0.991	0.988
Optimal threshold	0.638	0.600	0.464	0.265	0.490	0.084	0.101

Se, sensitivity; Sp, specificity; PPV, positive predictive value; NPV, negative predictive value; Acc., accuracy; and AUC, area under the curve.

**Table 7:** Performance of classifier implementation on task 3.

	LR	KNN	SVM	GNB	RF	XGB	Adaboost
Acc. (%)	74.41%	69.66%	74.14%	73.09%	71.50%	86.54%	77.84%
Se (%)	81.10%	74.39%	87.20%	71.33%	90.24%	79.88%	89.02%
Sp (%)	69.30%	66.05%	64.19%	74.15%	57.21%	91.63%	69.30%
PPV (%)	66.83%	62.56%	65.00%	62.58%	61.67%	87.92%	68.87%
NPV (%)	82.78%	77.17%	86.79%	81.02%	88.49%	85.65%	89.22%
AUC	0.841	0.759	0.837	0.787	0.802	0.911	0.857
Optimal threshold	0.336	0.400	0.266	0.028	0.359	0.468	0.218

Se, sensitivity; Sp, specificity; PPV, positive predictive value; NPV, negative predictive value; Acc., accuracy; and AUC, area under the curve.

**Table 8:** Performance of classifier implementation on task 4.

	LR	KNN	SVM	GNB	RF	XGB	Adaboost
Acc. (%)	76.99%	73.99%	77.22%	75.03%	77.91%	96.55%	90.56%
Se (%)	82.85%	63.32%	74.93%	64.12%	79.16%	94.20%	88.65%
Sp (%)	72.45%	82.24%	78.98%	83.47%	76.94%	98.37%	92.04%
PPV (%)	69.93%	73.39%	73.39%	75.00%	72.64%	97.81%	89.60%
NPV (%)	84.52%	74.35%	80.29%	75.05%	82.68%	95.63%	91.30%
AUC	0.848	0.809	0.847	0.786	0.869	0.994	0.963
Optimal threshold	0.326	0.600	0.397	0.999	0.398	0.597	0.434

Se, sensitivity; Sp, specificity; PPV, positive predictive value; NPV, negative predictive value; Acc., accuracy; and AUC, area under the curve.

**Figure 3:**
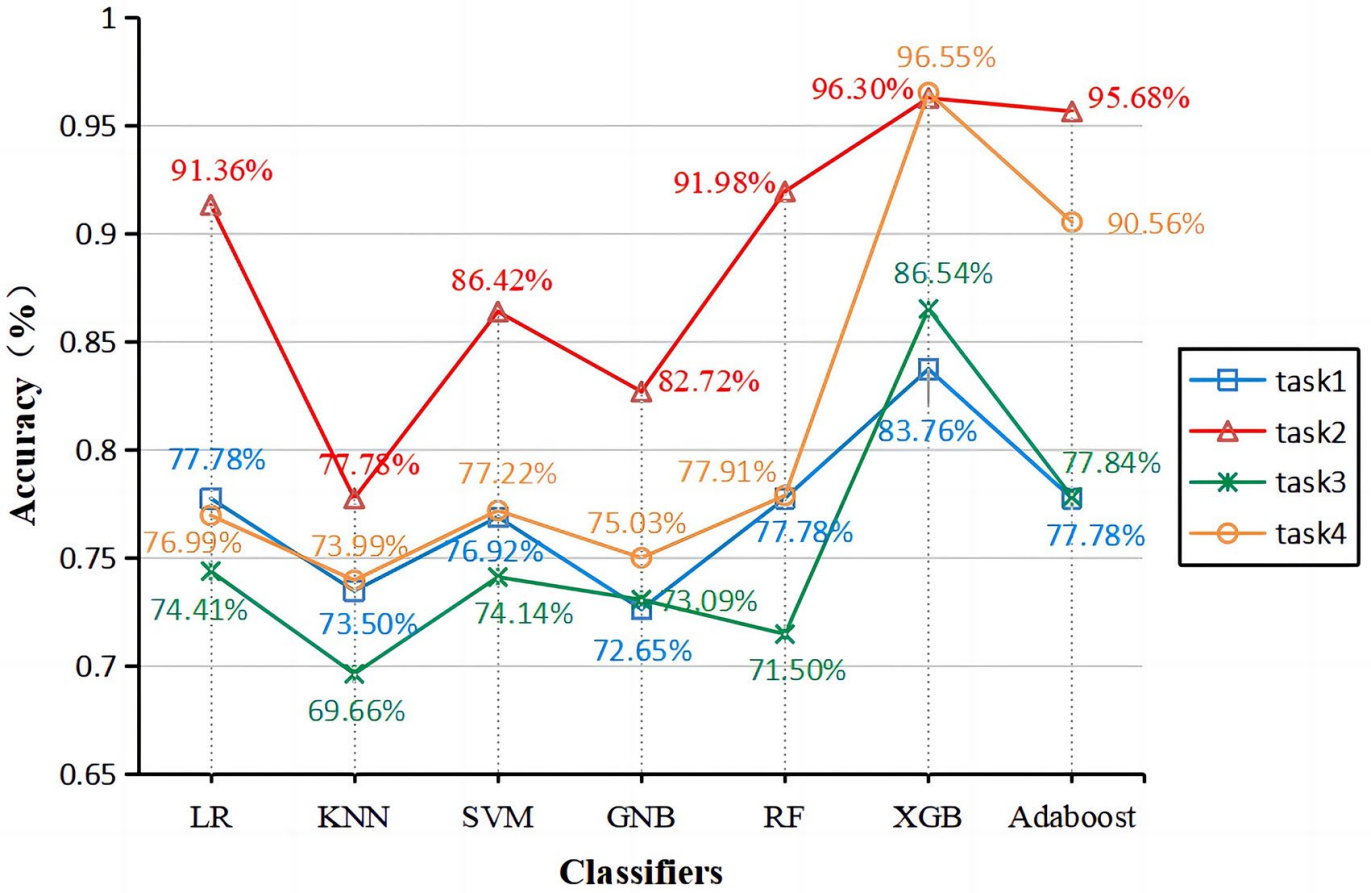
Accuracy of each classifier on four tasks. Task1 (blue line), Task2 (red line), Task3 (green line), and Task4 (orange line).

**Figure 4:**
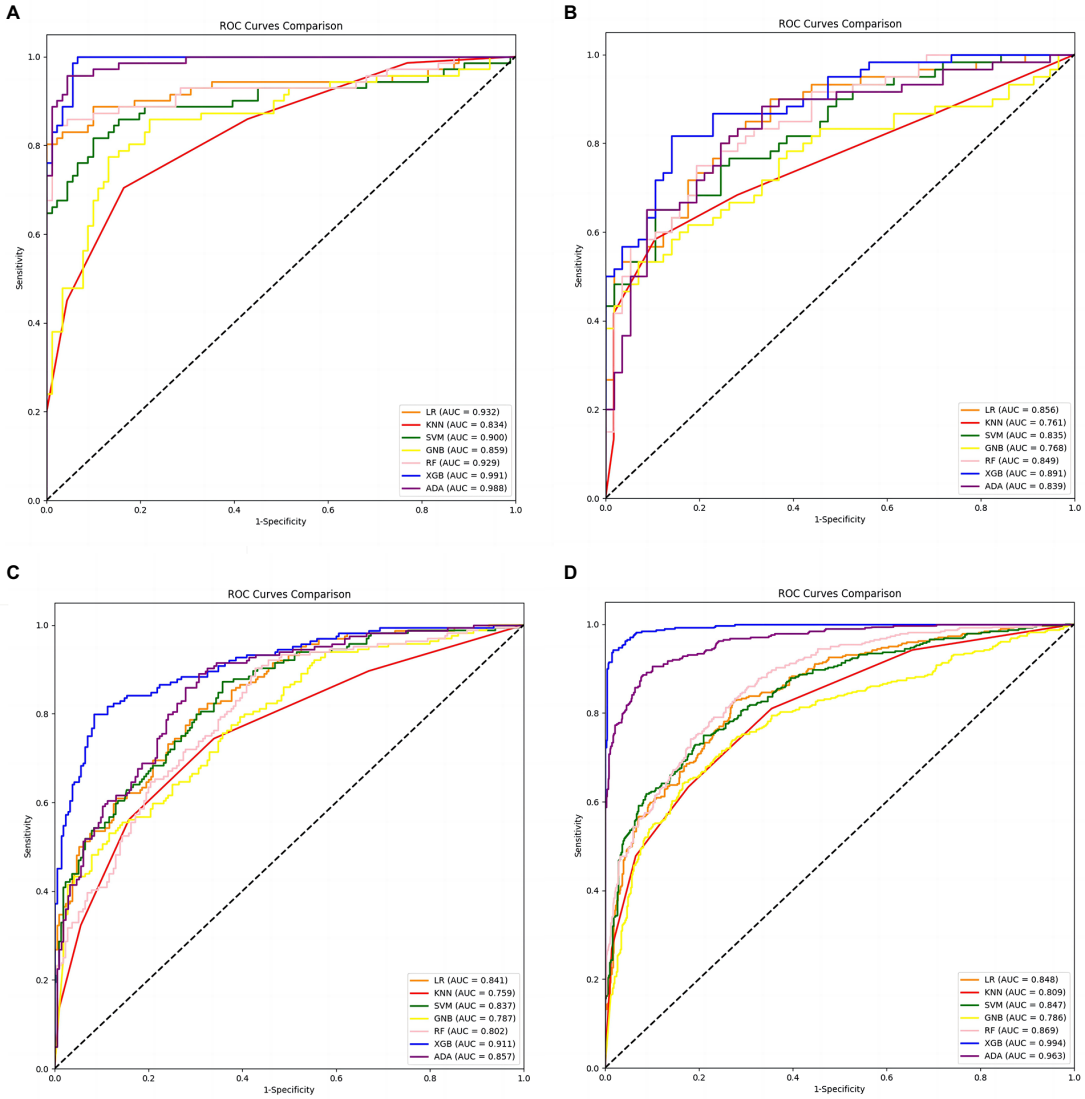
Receiver operating characteristic (ROC) curves were calculated to differentiate AD patients and healthy controls. **(A)** ROC curves for each classifier in Task 1. **(B)** ROC curves for each classifier in Task 2. **(C)** ROC curves for each classifier in Task 3. **(D)** ROC curves for each classifier in Task 4. The black dashed line indicates the performance of the random classifier.

Figures 3, 4 show that XGB performs the best in accuracy and AUC on each task among the seven classifiers. Among the two graphics class tasks, task 2 (copying intersecting pentagons) had better discrimination. Acc. = 96.30%, ROC curve analysis showed the best threshold of 0.084, sensitivity = 100%, specificity = 93.41%, PPV = 92.21%, NPV = 100%, and AUC = 0.991 (Table 6; Figure 4B). Among the two text-based tasks, task 4 (transcribing a sentence) showed better distinction with the highest accuracy. Acc. = 96.55%, ROC curve analysis showed the best threshold value of 0.597, sensitivity = 94.20%, specificity = 98.37%, PPV = 97.81%, NPV = 95.63%, and AUC = 0.994 (Table 8; Figure 4D).

## 4. Discussion

In this study, two graphic tasks and two text tasks were set. A significant correlation was found in a Spearman analysis between the patient group’s MoCA-BC scores and kinematic measures. This result was the same as in a previous study (Kawa et al., 2017). A significant correlation was found between cognitive level and kinematic writing parameters, indicating poorer motor coordination in patients with cognitive impairment.

The present study found that the average mean value of pressure would be higher in the AD group, even though the pressure of task 1 did not reflect a significant correlation, but still, the average mean value of pressure was somewhat higher in the AD group. This was entirely consistent with previous observational results that writing pressure became higher when writing in AD patients (Zhào et al., 2021). In contrast, some reports suggest that writing pressure in AD participants decrease rather than increase at (Werner et al., 2006). The increase in time in the air reflects the need for more thinking and reaction time during writing in the AD group (Yu and Chang, 2018). The higher number of slower speeds and slower accelerations in the AD group, as well as the higher entropy values for speeds and accelerations, indicate that the AD group has less stability in speed and acceleration during the writing and a decreased ability to keep writing speed stable (Schrter et al., 2003; Yan et al., 2008; Yu and Chang, 2018; Delazer et al., 2021). Although the handwriting characteristics of the two groups differed in the two types of tasks with different levels of difficulty, overall, the AD group would have higher writing pressure, slower writing speed, and reduced writing stability.

This study also observed an increase in the number of strokes in the AD group in the text task. This phenomenon may occur because some AD patients have a reduced ability to control hand movements, resulting in miswritten writing. Also, because some AD patients are unsure if they are writing correctly, they repeatedly trace text that has already been completed.

The present study found that not every task produced the same handwriting characteristics, and the classification efficacy of handwriting characteristics changed with different tasks. It was found that copying intersecting pentagons was more effective than connecting fixed points in the graphical task, probably because the task of copying pentagons was more complex and required more thinking and conceptualization by the participants. In the text task, transcribing a paragraph was more effective than dictating three simple words, probably because transcribing a paragraph was more complex and required more handwriting movements and thinking time, so it was hypothesized that increasing the number of words in the text task would help distinguish the AD group from the control group, but increasing the number of words would increase the participants’ burden, so the appropriate number of words in the text transcription task also needs further study. Overall complex and demanding writing tasks help to detect early AD, which had the same findings as previous studies (Schrter et al., 2003; Yan et al., 2008; Ghaderyan et al., 2018; Yu and Chang, 2018; Delazer et al., 2021).

Handwriting and drawing performance may be early indicators of brain dysfunction (Schrter et al., 2003; Garre-Olmo et al., 2017). Therefore, using kinematic parameters to assess fine motor function during handwriting and drawing may be helpful in clinical settings (Garre-Olmo et al., 2017). In situations where specialized physicians and specialized equipment are in short supply, such as community hospitals, handwriting analysis can assist general practitioners in performing rapid initial screening to ensure rapid referral of newly identified patients with possible AD to specialist clinics. Changes in handwriting characteristics or abnormal performance of AD patients reflect their physiological dysfunction to varying degrees, and the analysis and study of handwriting characteristics of AD patients to establish valid quantitative evaluation indicators. The analysis of handwriting characteristics of AD patients can provide an effective technical tool for clinical diagnosis, evaluation of the extent of the disease, and assessment of the efficacy of AD, and is also valuable for clinical research on AD movement disorders.

However, this study has certain limitations: the sample size is small, and the sample size can continue to be increased in the future. However, the current analysis was performed by splitting the writing task into single strokes with a small sample size and achieved the highest accuracy rate of 96.55%. However, it is believed that a higher accuracy rate will be obtained when the sample size is increased. Due to the differences in individual writing habits and abilities, this study only controlled for education level, habitual hand use, etc. No differences were found, and each individual’s writing habits and abilities can be divided in detail to improve the accuracy of the sample in the future. This study mainly collected cross-sectional data from patients with early AD, and it would be helpful to collect longitudinal data in the future to study the handwriting of AD patients. Although the differences in handwriting characteristics between AD patients and healthy patients were explored and analyzed, and a better classification model was constructed. This is not a substitute for professional diagnosis but only serves as an early warning or aid in diagnosis in the absence of professional doctors and equipment.

## 5. Conclusion

In this study, the handwriting characteristics of 34 AD patients and 45 healthy controls were collected and quantitatively analyzed using the self-developed “Handwriting characteristic analysis system of Huzhou University (huSaS),” which improves the efficiency of handwriting collection and analysis and provides the possibility of large-scale handwriting analysis. The study demonstrated that analyzing the differences in handwriting characteristics between the AD and control groups is promising in AD-assisted screening or assisted diagnosis.

The study found that there was a significant correlation between cognitive level and kinematic writing parameters in the AD and control groups, and the AD group had greater writing pressure, slower writing speed, and decreased writing stability. In the handwriting task with high complexity, the classifier classified better results, with the highest accuracy of 96.55%. These conclusions all support the hypothesis that handwriting analysis can assist in screening for AD.

Writing pressure is a controversial point. We found that the writing pressure would be greater in the AD group, which is similar to some previous studies, but this part needs further research. In terms of speed and writing stability, we conclude that AD patients will have slower writing speed, decreased writing stability, and poorer motor coordination, which is the same view as other researchers. At the same time, in terms of the effect of handwriting task difficulty on the classification effect, we draw a new view that the more difficult handwriting task is easier to distinguish between the two groups of people. In the future, we will try more complex writing tasks or combine handwriting features with graphics to expect better results.

This study provides new adjuvant screening modalities for early Alzheimer’s disease. The auxiliary diagnostic model can effectively screen for early Alzheimer’s disease and maintain high accuracy. Meanwhile, handwriting analysis also provides new ideas for early screening of other related diseases.

## Data availability statement

The raw data supporting the conclusions of this article will be made available by the authors, without undue reservation.

## Ethics statement

The studies involving human participants were reviewed and approved by the Medical Ethics Committee of the Third People’s Hospital of Huzhou, China (review number: 2022-049). The patients/participants provided their written informed consent to participate in this study.

## Author contributions

HQ, LW, and QL contributed to conception and design of the study. XT, KZ, ZW, and RZ organized the database. RZ and CZ performed the statistical analysis. HQ and RZ wrote the first draft of the manuscript. QL and CZ wrote sections of the manuscript. QL and HQ provided funding. All authors contributed to the article and approved the submitted version.

## Funding

This work was supported by the Chaomi S&T Company Cooperation Project (No. HK16003), the Scientific Research Fund of Zhejiang Provincial Education Department (No. Y202250185), and the Postgraduate Research and Innovation Project of Huzhou University (No. 2022KYCX36).

## Conflict of interest

XT was employed by the company Cloudbutterfly Technology Co., Ltd.

The remaining authors declare that the research was conducted in the absence of any commercial or financial relationships that could be construed as a potential conflict of interest.

## Publisher’s note

All claims expressed in this article are solely those of the authors and do not necessarily represent those of their affiliated organizations, or those of the publisher, the editors and the reviewers. Any product that may be evaluated in this article, or claim that may be made by its manufacturer, is not guaranteed or endorsed by the publisher.

## References

[ref1] Ammal S. M. Manoharan P. S. (2023). Multi-headed deep learning models to detect abnormality of Alzheimer? S patients. Comput. Syst. Sci. Eng. 44, 367–390. doi: 10.32604/csse.2023.025230

[ref2] Caterina Silveri M. Corda F. Di Nardo M. (2007). Central and peripheral aspects of writing disorders in Alzheimer's disease. J. Clin. Exp. Neuropsychol. 29, 179–186. doi: 10.1080/13803390600611351, PMID: 17365253

[ref3] Chen K. L. Xu Y. Chu A. Q. Ding D. Liang X. N. Nasreddine Z. S.. (2016). Validation of the Chinese version of Montreal cognitive assessment basic for screening mild cognitive impairment. J. Am. Geriatr. Soc. 64, e285–e290. doi: 10.1111/jgs.14530, PMID: 27996103

[ref4] Cilia N. D. Alessandro, T D. De Stefano C. Fontanella F. Molinara M. (2021). From online handwriting to synthetic images for Alzheimer's disease detection using a deep transfer learning approach. IEEE J. Biomed. Health Inform. 25, 4243–4254. doi: 10.1109/JBHI.2021.3101982, PMID: 34347614

[ref5] Cilia N. D. Alessandro, T D. De Stefano C. Fontanella F. (2022). Deep transfer learning algorithms applied to synthetic drawing images as a tool for supporting Alzheimer’s disease prediction. Mach. Vis. Appl. 33, 1–17. doi: 10.1007/s00138-022-01297-8

[ref6] Cilia N. D. Stefano C. D. Fontanella F. Molinara M. Freca A. S. D. (2019). “Handwriting analysis to support Alzheimer's disease diagnosis: A preliminary study” in *International Conference on Computer Analysis of Images and Patterns*.

[ref7] Delazer M. Zamarian L. Djamshidian A. (2021). Handwriting in Alzheimer's disease. J. Alzheimers Dis. 82, 727–735. doi: 10.3233/JAD-210279, PMID: 34057089

[ref8] Dentamaro V. Impedovo D. Pirlo G. (2021), "An analysis of tasks and features for neuro-degenerative disease assessment by handwriting."

[ref9] Edition F. (2013). Diagnostic and statistical manual of mental disorders. Am. Psychiat. Assoc. 21, 591–643.

[ref10] Evyapan Akku D. (2015). "The Ege agraphia test battery for identifying the writing disorders in cases with mild cognitive impairment and Alzheimer's disease/longshore JW." 27711939

[ref11] Folstein M. F. Folstein S. E. Mchugh P. R. (1975). Mini-mental state: a practical method for grading the cognitive state of patients for the clinician. J. Psychiatr. Res. 12, 189–198. doi: 10.1016/0022-3956(75)90026-6 1202204

[ref12] Forbes K. E. Shanks M. F. Venneri A. (2004). The evolution of dysgraphia in Alzheimer’s disease. Brain Res. Bull. 63, 19–24. doi: 10.1016/j.brainresbull.2003.11.005, PMID: 15121235

[ref13] Garre-Olmo J. Faúndez-Zanuy M. López-De-Ipiña K. Calvó-Perxas L. Turró-Garriga O. (2017). Kinematic and pressure features of handwriting and drawing: preliminary results between patients with mild cognitive impairment, Alzheimer disease and healthy controls. Curr. Alzheimer Res. 14, 960–968. doi: 10.2174/1567205014666170309120708, PMID: 28290244 PMC5735518

[ref14] Gaugler J. James B. Johnson T. Reimer J. Solis M. Weuve J.. (2022). 2022 Alzheimer's disease facts and figures. Alzheimers Dement. 18, 700–789. doi: 10.1002/alz.12638 35289055

[ref15] Ghaderyan P. Abbasi A. Saber S. (2018). A new algorithm for kinematic analysis of handwriting data; towards a reliable handwriting-based tool for early detection of Alzheimer's disease. Expert Syst. Appl. 114, 428–440. doi: 10.1016/j.eswa.2018.07.052

[ref16] Ghilardi M. F. Alberoni M. Rossi M. Franceschi M. Mariani C. Fazio F. (2000). Visual feedback has differential effects on reaching movements in Parkinson's and Alzheimer's disease. Brain Res. 876, 112–123. doi: 10.1016/S0006-8993(00)02635-4 10973599

[ref17] Hughes J. C. Graham N. Patterson K. Hodges J. R. (1997). Dysgraphia in mild dementia of Alzheimer's type. Neuropsychologia 35, 533–545. doi: 10.1016/S0028-3932(96)00102-9, PMID: 9106281

[ref18] Hunter D. S. (1974). The relationship of selected teacher behaviors to STUDENT achievement and to STUDENT attitude in the United States dependents schools, European Area, University of Southern California.

[ref19] Impedovo D. Pirlo G. (2018). Dynamic handwriting analysis for the assessment of neurodegenerative diseases: a pattern recognition perspective. IEEE Rev. Biomed. Eng. 99:1. doi: 10.1109/RBME.2018.2840679 29993722

[ref20] Kahindo C. El-Yacoubi M. A. Garcia-Salicetti S. Rigaud A. Cristancho-Lacroix V. (2018). Characterizing early-stage Alzheimer through spatiotemporal dynamics of handwriting. IEEE Signal Process. Lett. 25, 1136–1140. doi: 10.1109/LSP.2018.2794500

[ref21] Kawa J. Bednorz A. Stępień P. Derejczyk J. Bugdol M. (2017). Spatial and dynamical handwriting analysis in mild cognitive impairment. Comput. Biol. Med. 82, 21–28. oi: 10.1016/j.compbiomed.2017.01.004 28126631

[ref22] Kverno K. (2022). New treatment aimed at preventing Alzheimer's dementia. J. Psychosoc. Nurs. Ment. Health Serv. 60, 11–14. doi: 10.3928/02793695-20220324-02, PMID: 35510912

[ref23] Lei P. Ayton S. Bush A. I. (2021). The essential elements of Alzheimer’s disease. J. Biol. Chem. 296, 100–105. doi: 10.1074/jbc.REV120.008207 PMC7948403 33219130

[ref24] Luzzatti C. Laiacona M. Agazzi D. (2003). Multiple patterns of writing disorders in dementia of the Alzheimer type and their evolution. Neuropsychologia 41, 759–772. doi: 10.1016/S0028-3932(02)00328-7, PMID: 12631527

[ref25] Mckhann G. M. Knopman D. S. Chertkow H. Hyman B. T. Jack C. R. Jr. Kawas C. H.. (2011). The diagnosis of dementia due to Alzheimer’s disease: recommendations from the National Institute on Aging-Alzheimer’s association workgroups on diagnostic guidelines for Alzheimer's disease. Alzheimers Dement. 7, 263–269. doi: 10.1016/j.jalz.2011.03.005, PMID: 21514250 PMC3312024

[ref26] Pereira C. R. Pereira D. R. Rosa G. H. Albuquerque V. H. Weber S. A. Hook C.. (2018). Handwritten dynamics assessment through convolutional neural networks: an application to Parkinson's disease identification. Artif. Intell. Med. 87, 67–77. doi: 10.1016/j.artmed.2018.04.001 29673947

[ref27] Platel H. Lambert J. Eustache F. Cadet B. Dary M. Viader F.. (1993). Characteristics and evolution of writing impairmant in Alzheimer's disease. Neuropsychologia 31, 1147–1158. doi: 10.1016/0028-3932(93)90064-7, PMID: 8107977

[ref28] Schrter R. A. Mergl K. Bürger H. Hampel J. H. Mller (2003). Kinematic analysis of handwriting movements in patients with Alzheimer's disease, mild cognitive impairment, depression and healthy subjects. Dement. Geriatr. Cogn. Disord. 15, 132–142. doi: 10.1159/000068484, PMID: 12584428

[ref29] Small G. W. Rabins P. V. Barry P. P. Buckholtz N. S. Dekosky S. T. Ferris S. H.. (1997). Diagnosis and treatment of Alzheimer disease and related disorders: consensus statement of the American Association for Geriatric Psychiatry, the Alzheimer's Association, and the American Geriatrics Society. JAMA 278, 1363–1371. doi: 10.1001/jama.1997.03550160083043 9343469

[ref30] Smits E. J. Tolonen A. J. Cluitmans L. Gils M. V. Conway B. A. Zietsma R. C.. (2014). Standardized handwriting to assess bradykinesia, Micrographia and tremor in Parkinson's disease. PLoS One 9:e97614. doi: 10.1371/journal.pone.0097614, PMID: 24854199 PMC4031150

[ref31] Stefano C. D. Fontanella F. Impedovo D. Pirlo G. Di Freca A. S. (2018). Handwriting analysis to support neurodegenerative diseases diagnosis: a review. Pattern Recogn. Lett. 121, 37–45. doi: 10.1016/j.patrec.2018.05.013

[ref32] Thies W. Bleiler L. (2012). 2012 Alzheimer's disease facts and figures Alzheimer's Association∗. Alzheimers Dement. 8, 131–168. doi: 10.1016/j.jalz.2012.02.001, PMID: 22404854

[ref33] Tseng M. H. Cermak S. A. (1993). The influence of ergonomic factors and perceptual–motor abilities on handwriting performance. Am. J. Occup. Ther. 47, 919–926. doi: 10.5014/ajot.47.10.919, PMID: 8109612

[ref34] Werner P. Rosenblum S. Bar-On G. Heinik J. Korczyn A. (2006). Handwriting process variables discriminating mild Alzheimer's disease and mild cognitive impairment. J. Gerontol. Ser. B Psychol. Sci. Soc. Sci. 61, P228–P236. doi: 10.1093/geronb/61.4.P228, PMID: 16855035

[ref35] White L. Fishman P. Basu A. Crane P. K. Larson E. B. Coe N. B. (2019). Medicare expenditures attributable to dementia. Health Serv. Res. 54, 773–781. doi: 10.1111/1475-6773.13134, PMID: 30868557 PMC6606539

[ref36] Yan J. H. Rountree S. Massman P. Doody R. S. Li H. (2008). Alzheimer’s disease and mild cognitive impairment deteriorate fine movement control. J. Psychiatr. Res. 42, 1203–1212. doi: 10.1016/j.jpsychires.2008.01.006, PMID: 18280503

[ref37] Yu N. Y. Chang S. H. (2018). Characterization of the fine motor problems in patients with cognitive dysfunction—a computerized handwriting analysis. Hum. Mov. Sci. 65, 71–79. doi: 10.1016/j.humov.2018.06.006 29934222

[ref38] Zhào H. Zhang Y. Xia C. Liu Y. Huang Y. (2021). Digital handwriting analysis of characters in Chinese patients with mild cognitive impairment. J. Vis. Exp. 169:e61841. doi: 10.3791/61841 33779592

