# Supplementary material for: A study of auxiliary screening for Alzheimer’s disease based on handwriting characteristics
Source: Front Aging Neurosci. 2023 Mar 15;15:1117250. doi: 10.3389/fnagi.2023.1117250 (PMC10050722; doi:10.3389/fnagi.2023.1117250)
Supplement: Supplementary file 1 [file Table_1.DOCX]

Supplementary Material

A study of auxiliary screening for Alzheimer's disease based on handwriting characteristics

Hengnian Qi^1†^, Ruoyu Zhang^1†^, Zhuqin Wei^2^, Chu Zhang^1^, Lina Wang^2^, Qing Lang^3*^, Kai Zhang^4^, Xuesong Tian^5^

*** Correspondence:** Qing Lang: [02476@zjhu.edu.cn](mailto:02476@zjhu.edu.cn)

**Captions**

**Figure S1** Handwriting tasks


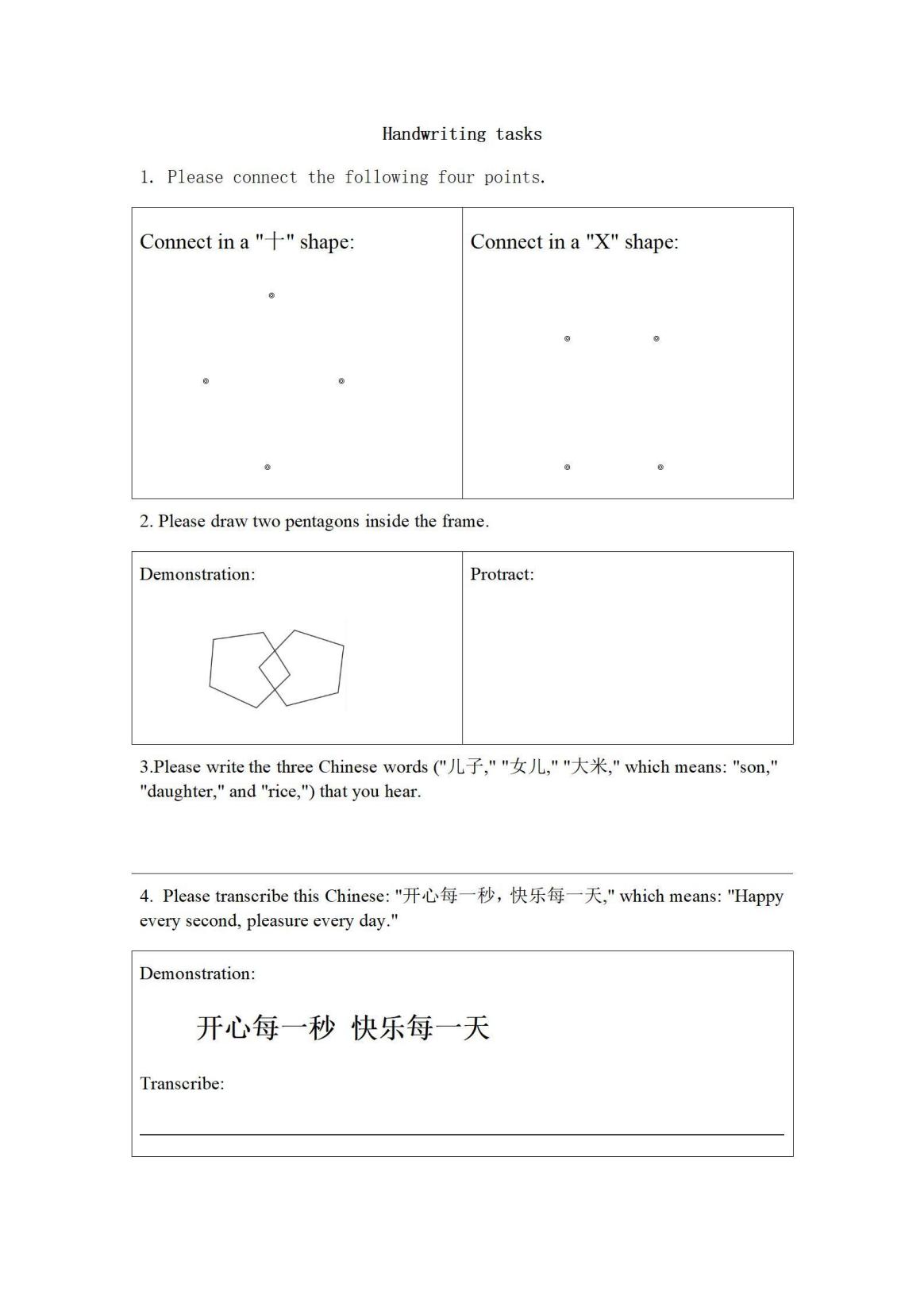


**Figure S1** Handwriting tasks
